# Multiseptate gallbladder coexisting with pancreaticobiliary maljunction treated by laparoscopic cholecystectomy: report of a pediatric case

**DOI:** 10.1186/s40792-022-01370-4

**Published:** 2022-01-21

**Authors:** Noboru Oyachi, Fuminori Numano, Keiichi Koizumi, Atsushi Takano, Hiroshi Shibusawa

**Affiliations:** 1 Department of Pediatric Surgery, Yamanashi Prefectural Central Hospital, 1-1-1 Kofu, Yamanashi, 409-8506 Japan; 2 Department of Surgery, Yamanashi Prefectural Central Hospital, Kofu, Japan; 3 Department of Pediatrics, Yamanashi Kosei Hospital, Yamanashi, Japan

**Keywords:** Multiseptate gallbladder, Pancreaticobiliary maljunction, Cholecystectomy, Ultrasonography, Pediatrics

## Abstract

**Background:**

A multiseptate gallbladder is a very rare congenital malformation in which the lumen is divided into variously sized multiseptal compartments. The pathogenesis and natural history of this disease remain uncertain. We herein describe a pediatric case of a multiseptate gallbladder with pancreaticobiliary maljunction (PBM), which was treated by laparoscopic cholecystectomy.

**Case presentation:**

A 5-year-old girl was referred to our hospital, because a multiseptate gallbladder had been incidentally detected on abdominal ultrasonography when she presented for transient abdominal pain. Ultrasonography showed hyperechoic septa throughout the lumen of the gallbladder, giving it a honeycomb appearance. The atrophied gallbladder had weak or no contractility. Magnetic resonance cholangiopancreatography performed to detect other coexisting biliary disorders revealed PBM without dilatation of the common bile duct. Although physical examination and laboratory tests revealed no abnormalities, we performed laparoscopic cholecystectomy to prevent cholecystitis and reduce the risk of cancer secondary to the PBM.

**Conclusions:**

In recent pediatric case reports, the indication and timing of cholecystectomy has tended to be determined by the patient’s symptoms and the presence of biliary complications. In the present case, however, the combination of a multiseptate gallbladder and PBM may become problematic in the future. Surgical treatment without delay was appropriate even in this pediatric patient.

## Background

A multiseptate gallbladder is a very rare congenital malformation of the gallbladder in which the lumen is divided into variously sized multiseptal compartments, forming a honeycomb appearance [1]. Only a few dozen cases have been reported in the English-language literature to date, and the number of pediatric cases is limited.

The development of a multiseptate gallbladder is occasionally associated with other biliary diseases. However, the pathogenesis and natural history of this disease remain unclear.

In this report, we describe a case involving a pediatric patient with a multiseptate gallbladder accompanied by pancreaticobiliary maljunction (PBM), which was treated by laparoscopic cholecystectomy.

## Case presentation

A 5-year-old girl was referred to our hospital, because a multiseptate gallbladder had been incidentally detected on abdominal ultrasonography when she presented for transient abdominal pain at the age of 4 years.

Her vital signs were normal, and physical examination revealed no abnormalities. Laboratory tests showed no elevation of the white blood cell count, and all serological findings, including liver function parameters and biliary enzymes, were within normal limits. The abdominal pain receded within a few days and was suspected to be due to constipation.

**Fig. 1:**
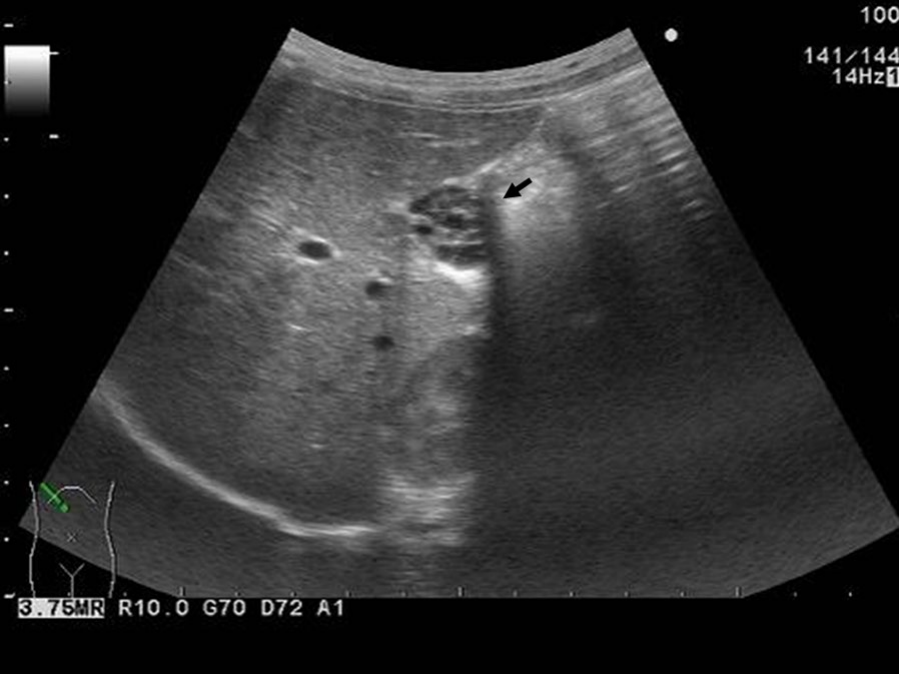
Abdominal ultrasonography. Abdominal ultrasonography showed multiple septa throughout the lumen of the gallbladder, giving it a honeycomb appearance (arrow). No gallstones or masses were identified

Ultrasonography showed hyperechoic septa throughout the lumen of the gallbladder, giving it a honeycomb appearance. The position of the gallbladder was normal, and there were no findings suggestive of a gallbladder stone or tumor formation; however, the wall was uneven. The atrophied gallbladder was 20 × 15 mm in size, and its size did not change during ultrasound follow-up; this suggested that the gallbladder had weak or no contractility (Fig. 1).

**Fig. 2:**
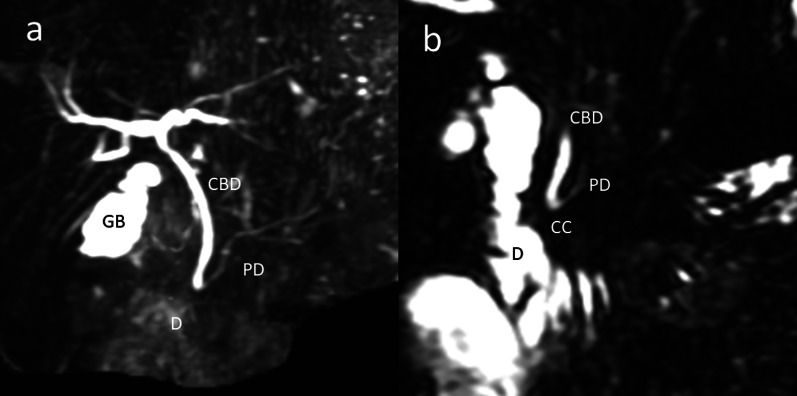
Magnetic resonance cholangiopancreatography. **a** Magnetic resonance cholangiopancreatography showed an uneven gallbladder wall and no dilatation of the intrahepatic bile duct or common bile duct. **b** However, magnetic resonance cholangiopancreatography showed pancreaticobiliary maljunction with a 1-cm common channel. *GB* gallbladder, *D* duodenum, *CBD* common bile duct, *PD* pancreatic duct, *CC* common channel

Magnetic resonance cholangiopancreatography was performed to search for other biliary and hepatic diseases. This imaging examination revealed a PBM with a 1-cm common channel without dilatation of the intrahepatic or common bile duct (Fig. 2).

Based on the imaging findings, the patient was diagnosed with a multiseptate gallbladder. In addition, the multiseptate gallbladder involved PBM without bile duct dilatation; therefore, cholecystectomy was recommended to prevent future cancer development in the gallbladder. We performed laparoscopic cholecystectomy when the patient was 5 years of age.

**Fig. 3:**
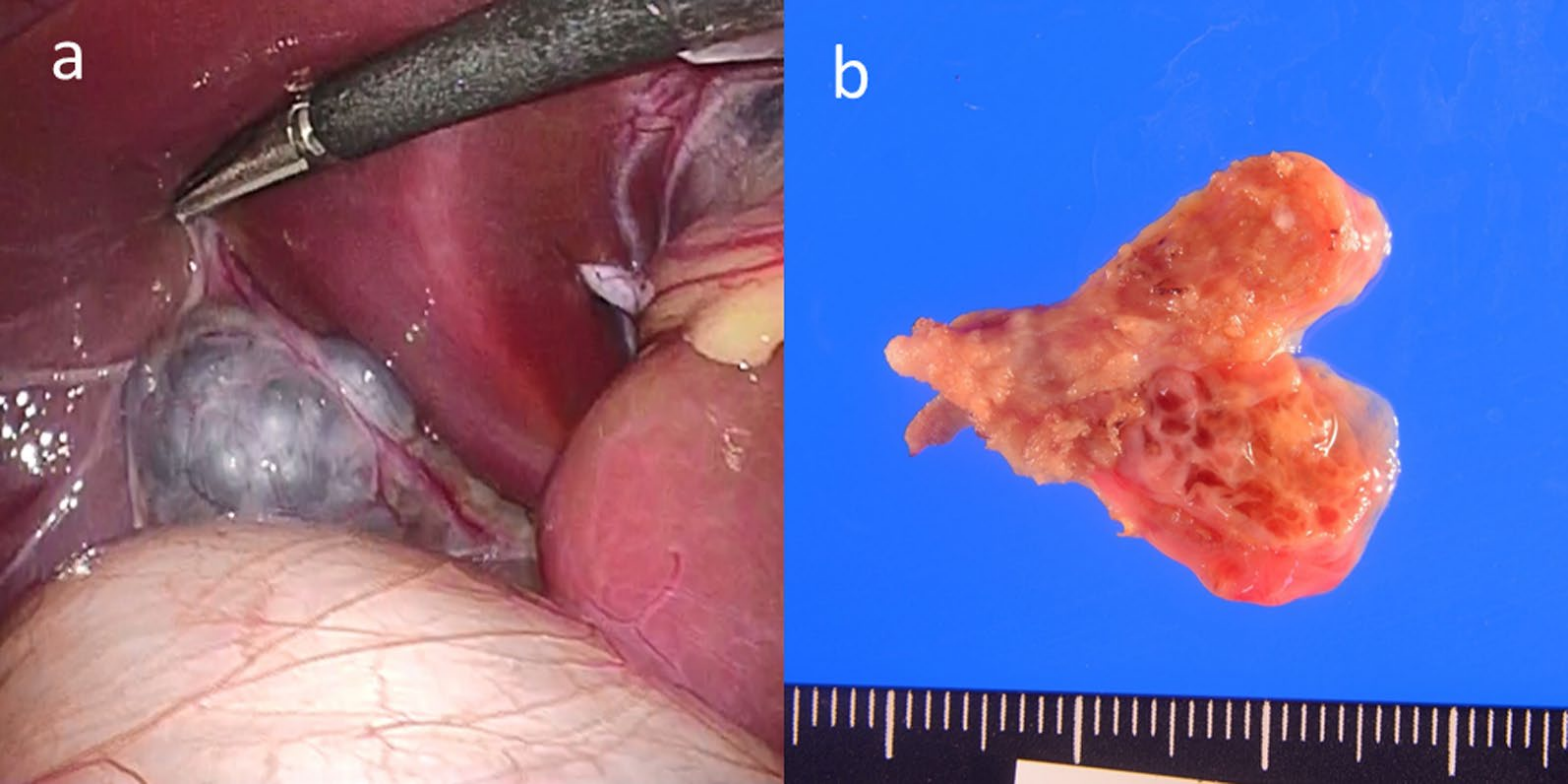
Operative findings and resected specimen. **a** Operative findings. The gallbladder was 20 mm in length and 15 mm in diameter with no adhesions to surrounding tissue. The surface of the gallbladder was uneven and had the appearance of a bunch of grapes. **b** The resected specimen. The gallbladder was soft and multiple septa were observed in the lumen, which was divided into variously sized compartments resulting in a honeycomb structure

The gallbladder was 20 mm in length and 15 mm in diameter with no adhesions to surrounding tissue. The surface of the gallbladder was uneven and had the appearance of a bunch of grapes (Fig. 3a). The cystic duct was partially excised, and insertion of a 5-Fr feeding tube was attempted for intraoperative cholangiography; however, the tube was unable to be passed, and no intraoperative images could be obtained. No bile leakage was observed at the incision site.

The resected gallbladder was soft and multiple septa were observed in the lumen, which was divided into variously sized compartments, resulting in a honeycomb structure (Fig. 3b). A small amount of brownish bile had collected in the gallbladder, and its biochemical profile was as follows: total bilirubin, 17.9 mg/dL; direct bilirubin, 16.9 mg/dL; and amylase, 3.0 IU/L. These findings indicated that the gallbladder had formed a microchannel with the cystic duct and that there was no reflux of pancreatic juice into the gallbladder.

**Fig. 4:**
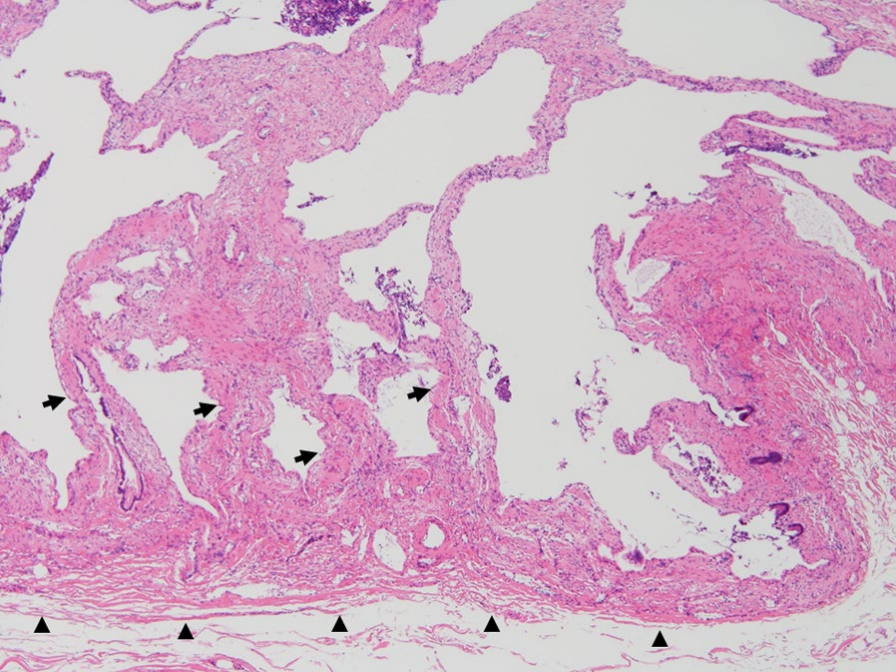
Pathological findings (hematoxylin–eosin stain, × 40). The septa were covered with normal mucosal epithelium, and the intrinsic muscular layer (arrow) continued from the gallbladder wall (arrowhead). There were no inflammatory changes or atypical cells in the epithelium

Microscopically, the septa were covered with normal mucosal epithelium, and the intrinsic muscular layer of the gallbladder wall continued to the septa. There were no inflammatory changes or atypical cells in the epithelium (Fig. 4).

The postoperative course was uneventful, and the patient was discharged on the third postoperative day. The patient thereafter developed no onset of abdominal pain associated with cholangitis, pancreatitis, or biliary dilatation due to PBM.

## Discussion

A multiseptate gallbladder is a quite rare congenital anomaly characterized by multiple septal structures in the gallbladder. It was first described clinically and histopathologically by Simon and Tandon in 1963 [1]. To the best of our knowledge, there have been 14 reports of pediatric cases of multiseptate gallbladder in 11 English-language literatures to date [2–12] (Table 1). However, abdominal ultrasonography has been increasingly indicated in pediatric patients with abdominal pain, and this disease is now being incidentally confirmed in children as in adults. Biliary complications of a multiseptate gallbladder, such as cholelithiasis, cholecystitis [8], and congenital biliary dilatation (CBD) [3, 6, 9], have also been previously reported.

**Table 1:** Pediatric cases of a multiseptate gallbladder in the English-language literatures

Author [Reference]	Year	Age	Sex	Biliary symptom	Coexisting biliary disease	Treatment
Haslam [2]	1966	15	F	Yes	None	Cholecystectomy
Pery [3]	1985	8	F	Yes	CBD	Cholecystectomy choledochoduodenostomy
Adear [4]	1990	12	F	None	None	Observation
Strauss [5]	1993	3	M	None	None	Not reported
Strauss [5]	1993	9	F	Yes	None	Not reported
Strauss [5]	1993	16	M	Yes	None	Not reported
Tan [6]	1993	14	F	Yes	CBD cholangitis	Extrahepatic bile duct resection hepaticojejunostomy
Saddik [7]	1998	10	M	None	None	Observation
Erdogmus [8]	2004	10	F	Yes	Cholelithiasis cholecystits	Cholecytectomy
Erdogmus [8]	2004	12	M	None	None	Not reported
Bahadir [9]	2006	15 days	M	Yes	CBD	Extrahepatic bile duct resection hepaticojejunostomy
Wanaguru [10]	2011	9 months	F	No	None	Observation
Germia [11]	2013	10	M	Yes	Biliary sludge	Observation
La Mendola [12]	2019	3	F	Yes	Biliary sludge	Cholecystectomy
Present case	2022	5	F	None	PBM without biliary dilatation	Cholecystectomy

*CBD* congenital biliary dilatation, *PBM* pancreaticobiliary maljunction

Although the development of a multiseptate gallbladder in the human embryo is not fully understood, the predominant theory is that the gallbladder bud grows faster than the gallbladder bed, causing “Phrygian cap” formation with multiple deflections and twists [7, 13]. The presence of multiple septa with a honeycomb appearance in the gallbladder is a characteristic imaging finding. The gallbladder has an uneven surface, resembling a bunch of grapes in appearance. Histopathological findings indicate that the septa dividing the gallbladder are covered with normal mucosal epithelium containing a muscular layer.

The present case involving a 5-year-old girl was unique in several respects other than the patient’s young age. First, magnetic resonance imaging revealed the coexistence of PBM without biliary dilatation. Second, the gallbladder was atrophied. There was no clear gallbladder contraction. However, no subjective symptoms suggestive of biliary disease were observed during the clinical course.

A multiseptate gallbladder can reportedly coexist with biliary tract diseases, including PBM and congenital biliary dilatation [6, 14]. PBM is a congenital anomalous biliary disease that is thought to be related to CBD or biliary tract malignancies, in which the pancreatic duct and bile duct join anatomically outside the duodenal wall. The common channel is abnormally long in PBM, allowing reciprocal reflux of pancreatic juices and bile. This reflux provokes a variety of pathologies, especially cholangitis, pancreatitis, and a high incidence of biliary tract cancer [15].

Based on the past pediatric reports shown in Table 1, the association of multiseptate gallbladder and CBD was present in 20% of pediatric cases. Hence, it is necessary to investigate associated abnormalities of the biliary tract by magnetic resonance cholangiopancreatography or computed tomography to determine the optimal treatment strategy. When considering PBM, it is necessary to distinguish whether this entity is associated with biliary dilatation. In cases of PBM with biliary dilatation (CBD), resection of the bile duct including the gallbladder is regarded as the standard surgical procedure. In the absence of biliary dilatation, however, prophylactic cholecystectomy is recommended because of the high possibility of gallbladder cancer, regardless of the presence or absence of symptoms. On the other hand, in the field of pediatric surgery, there is an opinion that extrahepatic bile duct resection is preferable after considering the long-term risk of carcinogenesis [15]. There is no fixed view on the prophylactic resection of the extrahepatic bile duct, because multiseptate gallbladder is a rare disorder in children, and so is PBM without biliary dilatation. Since this patient has experienced neither cholangitis nor pancreatitis, we considered that prophylactic extrahepatic bile duct resection and biliary reconstruction for the narrow bile duct should be avoided because of the possibility of postoperative complications such as stricture and biliary leakage.

In our case, the size of the gallbladder was small, and it did not change on repeat ultrasonography. Examination of the bile components obtained from the surgical specimen suggested that viscid bile could pass through the small openings in the septa, but the diameter of the duct was very small. We speculate that the ability to maintain the bile within the gallbladder was chronically disturbed and that the contractility of the gallbladder was weak, leading to impaired gallbladder development. Although this patient was asymptomatic, several previous reports have described patients with gallstone-like attack symptoms due to poor bile drainage or chronic inflammation induced by stagnation of bile in the gallbladder [16–18].

Because our patient had PBM and future carcinogenesis was a matter of concern, we chose to treat the patient surgically considering that she was 5 years old and physically able to undergo laparoscopic surgery safely. No reports in the English-language literature to date have described a malignant tumor in a multiseptate gallbladder. Some reports have recommended periodic follow-up without surgery in patients without severe biliary symptoms in childhood [10, 11]. However, the multiseptal structure of the gallbladder causes impaired bile excretion that can cause increased intracystic pressure and cholecystitis, which may lead to chronic abdominal pain. Symptomatic patients, even children, should immediately undergo cholecystectomy to alleviate their symptoms.

## Conclusions

In recent pediatric case reports, the indication and timing of cholecystectomy tends to be determined by the patient’s symptoms and the presence of biliary complications. In the present case, however, the combination of a multiseptate gallbladder and PBM may become problematic in the future. Although the patient underwent cholecystectomy, the PBM remains. Therefore, the patient should continue to be followed up periodically to check for the development of biliary and pancreatic symptoms. Further accumulation of cases is necessary for a better understanding of this disease.

## Data Availability

The data set supporting the conclusion of this article is included within the article.

## Abbreviations

PBM: Pancreaticobiliary maljunction
CBD: Congenital biliary dilatation
